# Opium dependence and the potential impact of changes in treatment coverage level: A dynamic modeling study

**DOI:** 10.34172/hpp.2021.29

**Published:** 2021-05-19

**Authors:** Hosein Rafiemanesh, Afarin Rahimi-Movaghar, Ali Akbar Haghdoost, Alireza Noroozi, Jaleh Gholami, Bita Vahdani, Amin Afshar, Mohammad Salehi, Koorosh Etemad

**Affiliations:** ^1^Department of Epidemiology, School of Public Health and Safety, Shahid Beheshti University of Medical Sciences, Tehran, Iran; ^2^Iranian National Center for Addiction Studies (INCAS), Tehran University of Medical Sciences, Tehran, Iran; ^3^Modeling in Health Research Center, Institute for Futures Studies in Health, University of Medical Sciences, Kerman, Iran; ^4^HIV/STI Surveillance Research Center, and WHO Collaborating Center for HIV Surveillance, Institute for Futures Studies in Health, Kerman University of Medical Sciences, Kerman, Iran; ^5^Assistant Professor of Psychiatry, Clinical Research Development Unit, 22 Bahman Hospital, Qazvin University of Medical Sciences, Qazvin, Iran; ^6^Department of Neurosciences and Addiction Studies, School of Advanced Technologies in Medicine, Tehran University of Medical Sciences, Tehran, Iran

**Keywords:** Opium, Opium dependence, Heroin dependence, Iran

## Abstract

**Background** : The most common drug, illegally used in Iran is opium. The treatment of people with substance use disorder is one of the most important strategies in reducing its burden. The aim of this study was to investigate the effect of different increasing and decreasing opium treatment coverage on the patterns of abstinence, transition to heroin dependence and mortality, over 30 years.

**Methods:** This study was a dynamic compartmental modeling conducted in three stages: 1) presenting a conceptual model of opium dependence treatment in Iran, 2) estimating model’s parameters value, and 3) modeling of opium dependence treatment and examining the outcomes for different treatment coverage scenarios. The input parameters of the model were extracted from the literature, and secondary data analysis, which were finalized in expert panels.

**Results:** The number of opium dependence will increase from 1180550 to 1522063 [28.93% (95% CI: 28.6 to 29.2)] over 30 years. With a 25% decrease in coverage compared to the status quo, the number of deaths will increase by 459 cases [3.28% (95% CI: 0.91 to 5.7)] in the first year, and this trend will continue to be 2989 cases [15.63% (95% CI: 13.4 to 17.9)] in the 30th year. A 25% increase in treatment coverage causes a cumulative decrease of heroin dependence by 14451 cases [10.1% (95% CI: 9.5 to 10.8)] in the first decade.

**Conclusion:** The modeling showed that the treatment coverage level reduction has a greater impact than the coverage level increase in the country and any amount of reduction in the coverage level, even to a small extent, may have a large negative impact in the long run.

## Introduction

Opioid abuse is one of the most serious health threats in the world. In 2018, 58 million people used opioids at least once in the last 12 months,^1^ and it is estimated that opioids have been implicated in 76% of drug use disorders related deaths.^2^ Opioid use and its dependence is one of the most serious health problems in Iran and opioid dependence in Iranian men is designated as the fifth disability-adjusted life year (DALY) and the third leading cause of life loss due to disability (YLD).^3^

The most common drug, illegally used in Iran is opium and with consumption of 42% of the world’s opium, Iran is the largest consumer of opium in the world (World Drug Report 2010). In spite of long history of opium cultivation and consumption in Iran, its cultivation is limited and almost eradicated in the last 50 years, but opium consumption is still widespread because of increased cultivation in Afghanistan.^4^

According to Iranian National Mental Health Survey (IranMHS) in 2011, the prevalence of 5-times or more of illegal opioid use over the past 12 months for people aged 15-64 was 3.0% and the last 12-month opioid use disorder was 2.2%.^5^ In Iran, more than 80% of opioid use and dependence of opioids belongs to opium and the most of Iranians drug dependence, have a history of opium use or dependence their life time.^5^

The treatment of people with substance use disorder is one of the most important strategies in reducing its burden of drug dependency. In Iran, Drug treatment policies have undergone many changes during the time and the start of methadone maintenance treatment (MMT) in 2004 and Buprenorphine maintenance treatment (BMT) in 2006 have been two of the most important turning points.^6^ Due to the necessity of drug use disorders treatments, their coverage is considered as of the indicators of United Nations Sustainable Development Goals for 2030.^7^ According to the WHO recommendations, treatment coverage (treatment entry) of opioid dependence less than 20%, 20%-40%, and more than 40% is considered as low, moderate, and high coverage level, respectively.^8^

The policy makers in each country need to estimate the impact of different interventions and treatment scenarios of the dependence population over the time and choose the best scenario. Dynamic models can be used to make estimations using scarce data, confirmation of hypotheses and predict consequences over time based on different scenario analysis.^9^

In spite of opioid dependence problem scale in Iran and the feasibility of provide therapeutic interventions for dependence prevention, rehabilitation or preventing negative consequences, no attempts have been made to develop dynamic models to investigate the impact of different opioid dependence treatment coverage scenarios. The current study is the first of its kind in Iran that conducted using dynamic modeling with the aim of investigating the effect of different increasing and decreasing opium treatment coverage on the patterns of abstinence, heroin dependence following opium dependence and mortality, over 30 year.

## Material and Methods

**Figure 1:**
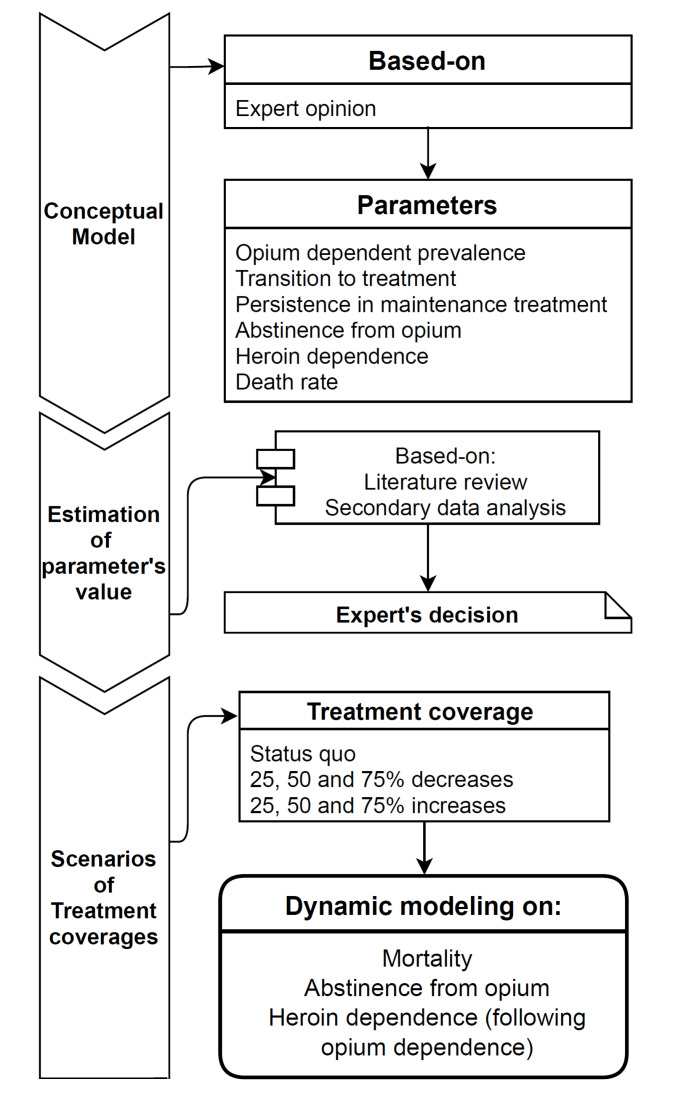
Flow diagram of study.

This study is a dynamic compartmental modeling that was conducted in three stages: 1) presenting a conceptual model of opium dependence treatment in Iran, 2) estimating model’s parameters value and 3) Modeling of opium dependence treatment and examining the outcomes for different treatment coverage scenarios. Figure 1 summarizes this study’s implementation process.

### Model description

The conceptual model of opium dependence treatment was designed based on the addiction experts’ opinions. In this study, opium dependence was considered as dependency on Opium and its derivatives (opium-shire and sookhteh). In this model, was considered two negative consequences including death and heroin dependence following opium dependence, and one positive consequence that was illegal opiate abstinence.

In this model, there are 4 paths in front of opium dependence:

- MMT
- BMT
- Other long-term treatments for relapse prevention (including psychological interventions, participation in self-help groups such as narcotics anonymous, oral or long-acting naltrexone) after detoxification
- Do not seeking treatment or nonevidence-based treatments (except of 3 above mentioned treatments).

**Figure 2:**
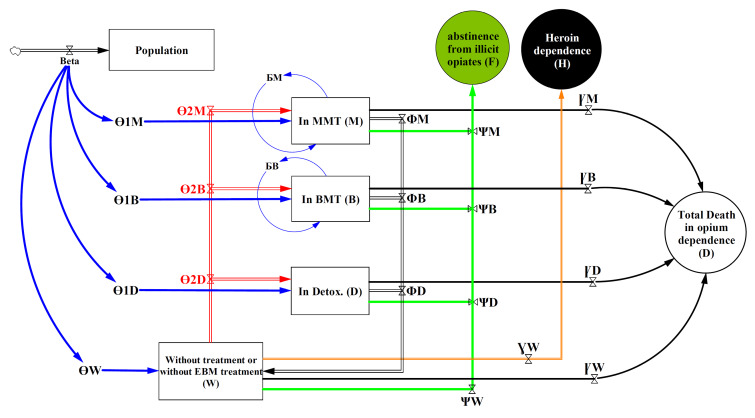
The dynamic modeling flow diagram of opium dependence treatment in Iran.

In the proposed model, if opium dependence treatment will be unsuccessful in each treatment group at the end of each year, people will be transition to untreated group and then the beginning of each year will enter to 3-evidence-based treatment groups or remain in untreated or nonevidence-based treatments group, based-on annual transition probability to each treatment group. It should also be noted that in this treatment model, for each of the four groups of the model stocks, there was a path for abstinence (treatment success) or death. Also, considered a path to heroin dependence following opium dependence of untreated group as a result of not entering evidence-based treatment (Figure 2).

Retention in maintenance treatment was considered to mean staying in treatment for one year with or without treatment success. Not taking any illegal opioids in the last month (12th month) was considered a success of the treatment. Also, the success of the treatment for the detoxification and relapse prevention group was considered in the sense of completing the treatment (detoxification and relapse prevention) and abstinence illegal opiate in the last month (12th month). Non-consumption can be confirmed by self-declaration or 2-4 negative urine tests in a row in the 12th month.

### Estimation of model parameters

To estimate the value of model parameters, we first consulted with experts about the existence of related research or data to obtain parameter values and in the absence of such data, literature review conducted to obtain parameter values.

In this study, secondary data analysis conducted for the data of the Iranian Drugs Abuse Treatment Information System (IDATIS)^10^ as well as the data of the last 3 years (2016-2018) of Iranian National Center for Addiction Studies (INCAS) treatment episode dataset (TEDS).^11^ The IDATIS is a web-based software for managing substance abuse treatment information in Iran, which has been developed in accordance with the MMT and BMT protocols to ensure the accurate implementation of the executive treatment instructions in Iran. This system launched in 2016, the system contains data on patients receiving maintenance treatment, and many reputable public and private centers across the country enter their client data in this system. Also, the TEDS data of the INCAS is the data of patients who referred to the clinic of this center who were referred and accepted due to problems related to a substance dependence.

The results of the RSA study (Rapid Situation Assessment of drug abuse in Iran) in 2018,^12^ the National Mental Health Survey,^5^ the follow-up study of opioid use and opioid use disorder,^13^ as well as the systematic review of mortality of opioid dependence in Iran^14^ were considered as needed to estimate the model parameters. The literature reviews were conducted specifically for retention in methadone and buprenorphine maintenance treatments, abstinence (treatment success) in different treatment groups, and death.

Due to the limitation of national evidence related to death rate by treatment groups (stocks), literature review was conducted based on systematic review studies on the death rate of drug dependence in the world^15,16^ and meta-analysis was conducted to obtain these values. In this meta-analysis, the annual mortality rate for non-injecting non-heroin dependence was performed and after obtaining the pooled annual mortality rate for groups and total dependence, the ratio of annual death rate for each of the model groups (stocks) to the total death rate was calculated in non-injectable non-heroin opioid dependence. Then, by taking into account these ratios and the total annual death rate obtained for opium dependence in Iran based on the secondary analysis of the INCAS systematic review, the death rate was estimated for maintenance treatment groups. Evans, E^17^ study was used to estimate the death rate in the detoxification and relapse prevention group in the same way as calculating the ratio of death in this group to the total death rate. The death rate in the out-of-treatment or non-evidence-based treatment groups were also estimated based on the total annual death rate of Iranian opium dependence by taking into account the death rate of treatment groups.

Finally, after analyzing and extracting data from available sources, in order to obtain accurate values of the parameters, each of the results was presented in expert panels alongside with their important aspects and limitations. The values of the parameters were modified based on the consensus of expert opinions for opium dependence population of the country and for the real (uncontrolled) conditions of Iran. These sessions (five three-hour sessions) were conducted as focus group discussions with the presence of eight expert (therapists, policy makers and researchers in the field of drug dependence). The secondary data analysis and meta-analysis was performed using STATA software version 14.2 (StataCorp, Texas, USA). The meta-analysis for mortality conducted based-on random-effects model and the pooled effect size for treatment subgroup were presented using the forest plot and tables. The heterogeneity of the preliminary studies was evaluated by I^2^ statistic. Additional information on how to estimate of the models’ parameters value is presented in Supplementary file 1.

Current opium treatment coverage level (Status quo) in Iran was estimated 28.5%. It was also estimated that 8.5% (2.42% of all opium dependence) of the total treatment coverage belonged to the detoxification and relapse prevention group. The share of MMT and BMT in the total maintenance treatments (26.08% of total) was 79.42% and 20.58%, respectively. Therefore, the share of MMT and BMT for all opium dependence in Iran was 20.71% and 5.37%, respectively. Table 1 shows the estimated final values for the models’ parameters. The table describes the population and variables used in the dynamic model of opium dependence treatment.^5,10-16,18-43^

**Table 1:** Parameters value used in opium dependence treatment model

**Variable**	**Symbols**	**Value: n, %**	References*
Number of populations in the base			^5^
Prevalence of opium dependence among population 15 to 64		2.06	
Number of opium dependent population estimated for the year 2019	Pop	1 180 553	
Annual transition probability to each treatment group			^5,10-12,18^
To MMT (M)	Ɵ_(1,2)M_	20.71	
To BMT (B)	Ɵ_(1,2)B_	5.37	
To detoxification and relapse prevention treatment (D)	Ɵ_(1,2)D_	2.42	
To without or non-evidence base medicine treatment (W)	Ɵ_W_	71.50	
Retention in maintenance treatment for one year			^18-25^
In MMT	Ƃ_M_	33.3	
In BMT	Ƃ_B_	30.0	
Transition to opium abstinence at the end of one year (success of treatment) in each treatment group			
In MMT	Ψ_M_	27.0	^26-35^
In BMT	Ψ_B_	26.0	
In detoxification and relapse prevention treatment	Ψ_D_	10.0	^29,36-43^
In those without treatment/with non-evidence-based treatment	Ψ_W_	1.0	
Annual probability of discontinuation or unsuccessful treatment and transition to non-treatment group			Calculate
In MMT	Φ_M_	65.93	
In BMT	Φ_B_	69.61	
In detoxification and relapse prevention treatment	Φ_D_	89.47	
Annual probability of death in each group			^14-16^
In MMT	Ꝩ_M_	0.77	
In BMT	Ꝩ_B_	0.39	
In detoxification and relapse prevention treatment	Ꝩ_D_	0.53	
In without treatment/ with non-evidence-based treatment	Ꝩ_W_	2.91	
Annual transition probability to heroin dependence			^11,13^
In without treatment/ with non-evidence-based treatment	Ɣ_W_	1.50	
Increasing the population of opium dependents due to population growth	Beta		Calculate

* References used or secondary analyzed and presented at the focus group discussion.

### Implement models for different treatment coverage level scenarios

The time change unit in the dynamic model was considered one year for all parameters. In this study, the target population was opium dependence aged 15-64 years and their initial number was estimated for 2019 (based on the population estimation of the Iranian National Statistics Center) and the prevalence estimated for this population. In this study, current treatment coverage level was estimated for the country and considered as the current treatment scenario (Status quo). Then, the effect of three scenarios of 25%, 50% and 75% decrease in coverage compared to the status quo and three scenarios of 25%, 50% and 75% increase in coverage compared to the status quo was modeled and compared on the two negative consequences of the model (death and heroin dependence following opium dependence) and a positive outcome (abstinence of illegal opiates) for a period of 30 years. In this study, the prevalence of opium dependence in Iran during 30 years was assumed to be constant and only the increase in the population of opium dependence as a result of population growth based on by the Iranian National Statistics Center estimation was considered as input to the model. Modeling and scenario building were performed using Vensim DSS 6.4E software.

## Results

**Figure 3:**
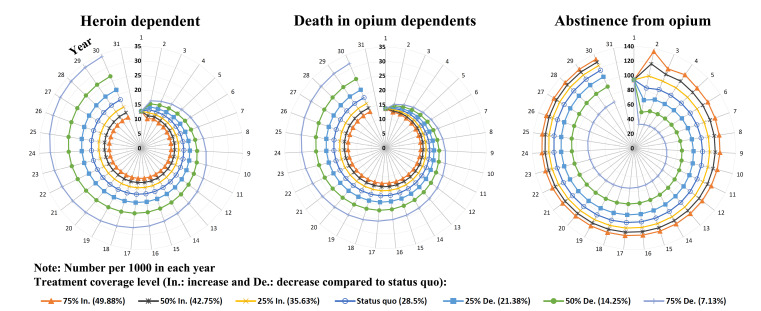
Number (per 1000) of heroin dependence (following opium dependence), death and abstinence from opium over 30-year dynamic modeling of opium dependent treatment by different coverage scenario in Iran.

The number of opium dependence will increase from 180 550 to 1 522 063 [28.93% (95% CI: 28.6 to 29.2)] over 30 years. In the current coverage level scenario (status quo), the number of deaths over 30 years will increase from 13 592 in the first year to 18 942 in the 30th year. Also, during the 30 years, the number of people transition to heroin dependence had will increase from 93 789 to 117 385, and the number of abstinences had will increase from 12 661 to 18 084. With an increase of 25%, 50% and 75% in the level of coverage treatment compared to the current coverage level (28.5%), the coverage level to 35.63%, 42.75% and 49.88%, respectively, and with a decrease of 25%, 50% and 75% in the coverage level of this amount reaches 21.38%, 14.25% and 7.13%. Figure 3 shows the number of heroin dependence, deaths and abstinence over 30 years for the current coverage level and also increasing and decreasing coverage level scenarios.

**Figure 4:**
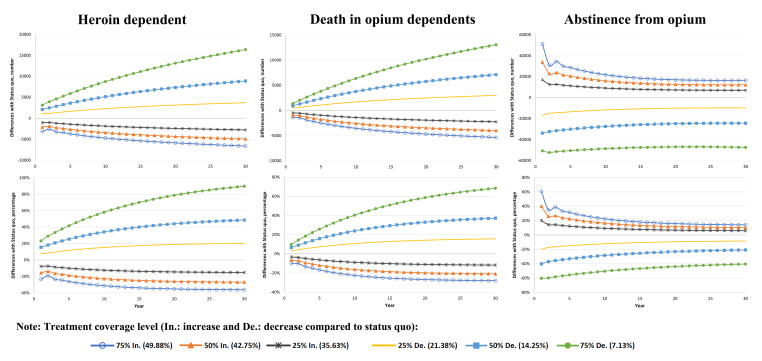
Changes in heroin dependence (following opium dependence), death and abstinence from opium in different treatment coverage level scenarios compared to baseline scenario.

With a 25% increase in treatment coverage compared to the status quo, the number of heroin dependence will decrease by 1,042 cases [7.76% (95%CI: 5.5 to 10.0)] in the first year, and this trend will continue to be 2771 cases [15.18% (95%CI: 13.4 to 17.0)] in the 30th year. In the scenario of 50% and 75% increase in coverage, the number of cases will decrease by 2083 cases [15.51% (95% CI: 13.4 to 17.6) and 3146 cases [23.43% (95% CI: 21.5 to 25.4)] in the first year, and will decrease by 4909 cases [26.90% (95% CI: 25.3 to 28.5)] and 6617 cases [36.26% (95% CI: 34.8 to 37.7)] in the 30th year, respectively. With a 25% decrease in treatment coverage compared to the status quo, the number of heroin dependence will increase by 1041 cases [7.75% (95% CI: 5.3 to 10.3)] in the first year, and this trend will continue to be 3713 cases [20.34% (95% CI: 18.0 to 22.7)] in the 30th year. In the scenario of 50% and 75% decrease in coverage, the number of cases will increase by 2083 cases [15.51% (95% CI: 12.9 to 18.2)] and 3124 cases [23.26% (95%CI: 20.5 to 26.1)] in the first year, and will increase by 8874 cases [48.63% (95% CI: 45.9 to 51.4)] and 16 389 cases [89.81% (95% CI: 86.4 to 93.2)] in the 30th year, respectively (Figure 4).

With a 25% increase in treatment coverage compared to the status quo, the number of deaths will decrease by 460 cases [3.28% (95% CI: 1.0 to 5.5)] in the first year, and this trend will continue to be 2,239 cases [11.71% (95% CI: 9.9 to 13.5)] in the 30th year. In the scenario of 50% and 75% increase in coverage, the number of cases will decrease by 919 cases [6.56% (95% CI: 4.3 to 8.8)] and 1385 cases [9.89% (95% CI: 7.7 to 12.0)] in the first year, and will decrease by 3973 cases [20.79% (95% CI: 19.1 to 22.5)] and 5352 cases [28.0% (95% CI: 26.4 to 29.6)] in the 30th year, respectively. With a 25% decrease in treatment coverage compared to the status quo, the number of deaths will increase by 459 cases [3.28% (95% CI: 0.91 to 5.7)] in the first year, and this trend will continue to be 2,989 cases [15.63% (95% CI: 13.4 to 17.9)] in the 30th year. In the scenario of 50% and 75% decrease in coverage, the number of cases will increase by 919 cases [6.56% (95% CI: 4.1 to 9.0)] and 1,379 cases [9.84% (95% CI: 7.4 to 12.4)] in the first year, and will increase by 7118 cases [37.24% (95% CI: 34.7 to 39.8)] and 13,081 cases [68.44% (95% CI: 65.5 to 71.4)] in the 30th year, respectively (Figure 4).

With a 25% increase in treatment coverage compared to the status quo, the number of abstinences will increase by 16 925 cases [20.09% (95% CI: 19.0 to 21.2)] in the first year, and by 6983 cases [5.95% (95% CI: 5.1 to 6.8)] in the 30th year. In the scenario of 50% and 75% increase in coverage, the number of cases will increase by 33 840 cases [40.18% (95% CI: 39.0 to 41.4)] and 51 198 cases [60.79% (95% CI: 59.5 to 62.1)] in the first year, and will increase by 12 150 cases [10.35% (95%CI: 9.5 to 11.2)] and 16 462 cases [14.02% (95% CI: 13.2 to 14.9)] in the 30th year, respectively. With a 25% decrease in treatment coverage compared to the status quo, the number of abstinences will decrease by 16 916 cases [20.08% (95% CI: 19.3 to 20.9)] in the first year, and by 9 802 cases [8.35% (95% CI: 7.6 to 9.1)] in the 30th year. In the scenario of 50% and 75% decrease in coverage, the number of cases will decrease by 33 839 cases [40.18% (95% CI: 39.5 to 40.8)] and 50 754 cases [60.26% (95% CI: 59.8 to 60.8)] in the first year, and will decrease by 24 392 cases [20.78% (95% CI: 20.1 to 21.4)] and 47 442 cases [40.42% (95% CI: 39.9 to 41.0)] in the 30th year, respectively (Figure 4).

**Figure 5:**
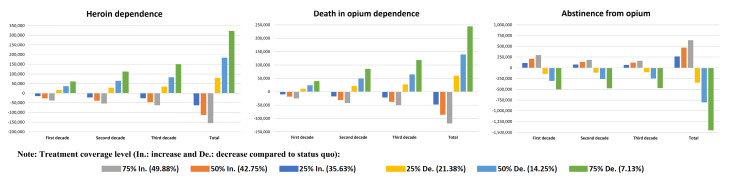
Cumulative number of heroin dependence (following opium dependence), death and abstinences from opium of three decades and total 30-year by different treatment coverage scenarios in Iran

Cumulative changes heroin dependence following opium dependence and death in different coverage scenarios in the second and third decades will have a greater impact than the first decade (Figure 5). A 25% increase in treatment coverage causes a cumulative decrease of heroin dependence by 14 451 cases [10.1% (95% CI: 9.5 to 10.8)] in the first decade and decrease by 26 187 cases [14.9% (95% CI: 14.4 to 15.5)] in the third decade. Cumulative changes in death with a 25% increase in treatment coverage resulted in a cumulative decrease of 9605 cases [6.4% (95% CI: 5.8 to 7.1)] in the first decade and a decrease of 21 046 cases [11.5% (95% CI: 10.9 to 12.1)] of death in the third decade. While cumulative changes in abstinence in different coverage scenarios in the first decade will have a greater impact than in the second and third decades. For example, a 25% increase in treatment coverage causes a cumulative increase of 113 939 cases [12.6% (95% CI: 12.2 to 12.8)] in the first decade and an increase of 70 298 cases [6.2% (95% CI: 6.0 to 6.5)] of abstinence in the third decade (Table 2).

**Table 2:** Number (sum of relative change) of cumulative changes in deaths, heroin dependence following opium dependence, and abstinence over three decades with changes in treatment coverage level relative to current coverage level

	**Treatment coverage level (%)**					
	**+25 (35.63%)**	**+50 (42.75%)**	**+75 (49.88%)**	**-25 (21.38%)**	**-50 (14.25%)**	**-75 (7.13%)**
Heroin dependence, n (Ɵ*)						
First decade	-14,451 (-1.01)	-26,885 (-1.88)	-37,820 (-2.65)	16,949 (1.18)	37,020 (2.58)	61,029 (4.25)
Second decade	-21,935 (-1.38)	-39,516 (-2.48)	-53,982 (-3.39)	27,928 (1.75)	64,227 (4.03)	112,586 (7.06)
Third decade	-26,187 (-1.49)	-46,552 (-2.66)	-62,906 (-3.59)	34,696 (1.98)	82,173 (4.69)	149,781 (8.54)
Mortality, n (Ɵ*)						
First decade	-9,605 (-0.64)	-17,921 (-1.19)	-25,218 (-1.68)	11,200 (0.74)	24,368 (1.62)	40,000 (2.65)
Second decade	-17,104 (-1.02)	-30,931 (-1.85)	-42,313 (-2.54)	21,588 (1.29)	49,343 (2.95)	85,875 (5.14)
Third decade	-21,046 (-1.15)	-37,493 (-2.04)	-50658 (-2.76)	27,739 (1.51)	65,412 (3.56)	118,560 (6.45)
Abstinence from opium, n (Ɵ*)						
First decade	113,939 (1.26)	210,081 (2.33)	294,956 (3.27)	-136,191 (-1.50)	-301,044 (-3.31)	-502,722 (-5.52)
Second decade	78,494 (0.77)	137,081 (1.34)	184,774 (1.81)	-107,179 (-1.05)	-257,878 (-2.52)	-475,200 (-4.65)
Third decade	70,298 (0.62)	122,070 (1.08)	164,843 (1.46)	-98,697 (-0.88)	-244,509 (-2.17)	-470,733 (-4.18)

* Ɵ is sum of relative change ([B-A]/A) for ten year. Note: The percentage change for each decade is Ɵ×10.

## Discussion

This study is the first study in the field of modeling the treatment of opium dependence in Iran. The aim of this study was to estimate the parameters of opium dependence based on research and experts’ opinion, for modeling different treatment coverage scenarios effects on abstinence, heroin dependence following opium dependence and mortality of opium dependence in Iran.

Although opium dependence accounts for a large portion of Iran’s total opioid dependence, and about two-thirds of dependents and treatment centers’ clients are opium dependences or have experienced opium use at some point in their lives,^5,44^ but few national and international studies have specifically addressed the treatment parameters of opium dependence.^25,40^ In this study, the parameters values were estimated based on the available evidences and was presented an opium dependence treatment model. In this study, we did not aim to estimate Iran’s opium dependence population which is affected by various factors like anti-drug trafficking policies and country’s health policies. So, we considered the prevalence of opium dependence as a constant rate (2.06%) for the general population over a period of 30 years, to be able to assess the impact of different scenarios on the level of treatment coverage. Of course, it should be mentioned that the effect of population growth on country’s opium dependence population was taken into account.

In current study, opium dependence prevalence in the population aged 15-64 years estimated to be about 2% and the treatment coverage for all of 3 evidence-based treatment groups (MMT, BMT and relapse prevention after detoxification) was estimated at 28.5%. According to the WHO recommendation, 20%-40% of OAT (opioid substitution therapy) coverage for all opioid dependence (injectable and non-injectable) is a moderate coverage level and less than 20 and more than 40, are low and high respectively.^8^ So, it seems that Iran’s current opium dependence treatment coverage level is at a medium or medium to low level and it is necessary to improve this situation. Therefore, considering the scenario of 25% increase (35.6%) and 50% increase (42.7%) in the treatment coverage level, we will probably reach the medium and high coverage levels.

The effect of 3 decreasing and 3 increasing scenarios on treatment coverage level was investigated. The results of the modeling showed that treatment coverage level reduction has a greater effect than the coverage level increase in the community and with a 25% decrease in treatment coverage level compared to the baseline scenario (28.5% coverage level) death and heroin dependence will increase by 7.4% and 11.8% in the first decade, respectively, and 15.0% of abstinence will decrease. In contrast, a 25% increase in coverage rate would result in a 6.4% reduction in death, a 10.1% decrease in heroin dependence, and a 12.6% increase in abstinence. Comparison of scenarios showed that treatment coverage level decrease affects the consequences more than its increase. Therefore, increasing the level of treatment coverage for communities where this amount is low as one of the effective interventions can be the chosen by policy makers in the field of dependence.

The results showed that a small percentage increase (25%) in the treatment coverage level could prevent 62 573 cases of heroin dependence following opium dependence and 47 755 deaths in the country during 30 years. Other studies, including the dynamic modeling study in Ukraine, have shown that increasing OAT capacity is cost-effective intervention.^45^ A mathematical modeling study has shown that OAT treatment coverage scaled up to 40%, including in prisons, compared to the absence of OAT, can reduce deaths by 7.7 percent in Kentucky, 10.7 percent in Kiev and 25.9 percent in Tehran over 20 years. In this study, the greater deaths reduction impact in Tehran was attributed to the larger population of injection drug users, more HIV positive cases in prisons and the reduction of HIV deaths in this group.^46^

The effect of the coverage level increase has also been investigated in similar issues such as alcohol consumption. A study in the UK has shown that increasing treatment coverage can have a significant positive effect on reducing mortality as well as reducing DALY and morbidity attributed to alcohol consumption.^47^

To increase of the treatment coverage level or increase the percentage of admission to treatment, in addition to the availability of services, other factors such as the cost of services, the dependents’ demand for treatment and the provision of high quality and effective services should be considered and the necessary actions should be taken by policy makers.^48,49^

The opium dependence in Iran is one of the main routes of dependence to other opioids like heroin which injecting drug user is one of its subsequent problems.^50,51^ policymakers’ attention is very important to increasing the level of treatment coverage and paying enough attention to prevent any decrease in current level of treatment coverage of opium dependence.

In Iran, over the last decade, the increase in treatment coverage was facilitated by the licensing of physicians to set up private dependence treatment centers. But due to the factors like difficulty of monitoring the private sector, concerns about the quality of services and the leakage of controlled substances such as methadone into the black market, the process of coverage increases stopped and in some cases the necessity of decreasing of dependence rehabilitation centers was proposed. However, the results of the present study show that any reduction in coverage level, even to a small extent, can have a large negative impact in the long run. Of course, it should be mentioned that this effect was evaluated only in the presence of a constant prevalence, and if the prevalence increases during the year, the impact of the negative consequences of reducing the coverage level will be more severe.

One of the advantages of the present modeling study was determining the values of the parameters based on various evidence (published and unpublished) and also using the opinion of experts to correct the parameters and bring their value closer to the reality of the opium dependence in Iran. One of the limitations of this study was the assuming opium dependence prevalence constant in the population over 30 years and also not considering other policies and variables affecting the population of opium dependence in Iran. However, other medical and non-medical policies such as insurance coverage, the fight against drug trafficking, as well as socio-economic policies and changes can have a different impact on the prevalence as well as the entry into treatment and the success of treatment. So, considering these variables in modeling can make the results of scenarios more accurate and closer to reality. The present study is the first study conducted in this field by the dynamic modeling method, that subsequent studies can answer more questions in this field by removing its limitations. This study is probably one of the first studies that has tried to estimate the parameters of opium dependence treatment. However, further primary studies in this area can reveal the accuracy of the estimates obtained in this study. For this purpose, studies at the level of national generalizability are proposed so that we can have more accurate values of these parameters. It is also suggested that future studies consider the cost-effectiveness of each scenario.

The results of this study help the country’s addiction policy makers to consider the overall impact of changes in the treatment level coverage of opium dependence for a long period in Iran and to consider them in their decisions. However, this study, like other studies of dynamic models, is along with assumptions that the results must always be interpreted with these assumptions in mind.

## Conclusion

The present study showed that due to population growth even in a constant prevalence of opium addiction, the trend of opium dependence will be increasing over 30 years. Therefore, at the status quo level of treatment coverage, will also increase the rate of heroin dependence following opium dependence and death. The modeling showed that treatment coverage level reduction has a greater effect than the coverage level increase in the country and any reduction in coverage level, even to a small extent, can have a large negative impact in the long run. Also, the effect of changes in treatment coverage levels on heroin dependence, following opium dependence, as well as death in the second and third decades will be more than in the first decade, while the effect on abstinence from opium will be more in the first decade.

## Acknowledgements

The authors wish to thank all members of the research team and others who facilitated this study.

## Funding

This paper was supported by the Shahid Beheshti University of Medical Sciences Research Council.

## Competing interests

The authors have no conflicts of interest to declare for this study.

## Ethical approval

Ethical approval was obtained from the Shahid Beheshti University of Medical Sciences Ethics Committee (No. IR.SBMU.PHNS.REC.1397.126).

## Authors’ contributions

The research idea proposed with the HR, AR and KE. The sections of theoretical framework and methodology completed with HR, AR, KE and AAH. The literature review and secondary data analysis conducted with HR, under supervision of KE and AR. The HR, AR, KE, AN, JG, BV, AA, and MS contributed in focus group discussion and parameter estimation. The method and analysis completed with HR, AR, KE and AAH. All authors discussed the results, implications and commented on the manuscript at all stages

## Supplementary materials


Supplementary file 1. Estimation of model parameter’s values.

## References

[1] United Nations Office on Drugs and Crime (UNODC). World Drug Report, 2020. Available from: http://www.unodc.org/wdr2020. Accessed September 5, 2020.

[2] United Nations Office on Drugs and Crime (UNODC). World Drug Report 2018. Available from: https://www.unodc.org/wdr2018. Accessed September 5, 2020.

[3] Forouzanfar MH, Sepanlou SG, Shahraz S, Dicker D, Naghavi P, Pourmalek F (2014). Evaluating causes of death and morbidity in Iran, global burden of diseases, injuries, and risk factors study 2010. Arch Iran Med.

[4] Aliverdinia A, Pridemore WA (2008). An overview of the illicit narcotics problem in the Islamic Republic of Iran. European Journal of Crime Criminal Law and Criminal Justice.

[5] Amin-Esmaeili M, Rahimi-Movaghar A, Sharifi V, Hajebi A, Radgoodarzi R, Mojtabai R (2016). Epidemiology of illicit drug use disorders in Iran: prevalence, correlates, comorbidity and service utilization results from the Iranian Mental Health Survey. Addiction.

[6] Ekhtiari H, Noroozi A, Farhoudian A, Radfar SR, Hajebi A, Sefatian S (2020). The evolution of addiction treatment and harm reduction programs in Iran: a chaotic response or a synergistic diversity?. Addiction.

[7] United Nations. Transforming our World: The 2030 Agenda for Sustainable Development. New York: United Nations; 2015.

[8] WHO, UNODC, UNAIDS. Technical guide for countries to set targets for universal access to HIV prevention, treatment and care for injecting drug users – 2012 revision. Available from: http://www.who.int/hiv/pub/idu/targets_universal_access/en/. Accessed September 5, 2017.

[9] Rossi C (2002). The role of dynamic modelling in drug abuse epidemiology. Bull Narc.

[10] Ministry of Health and Medical Education. Iran Substance Abuse Treatment Information System, IDATIS. Available from: https://idatis.behdasht.gov.ir.

[11] INCAS-TEDS. Iranian National Center for Addiction Studies (INCAS) Treatment Episode Dataset: TEDS. 2017-2019 (Unpublished data).

[12] Rafiey H, Narenjiha H, Alipour F. Rapid Situation Assessment of Drug Abuse in Iran, 2018. Department of Research and Education, Drug Control Headquarters, Presidency of the IR of Iran; 2020 (Unpublished data).

[13] INCAS. Outcome of opioid use and opioid use disorder; a six-year follow-up. 2019 (Unpublished data).

[14] Gholami J, Baheshmat Sh, Rostam-Abadi Y, Rahimi-Movaghar A. Drug-related deaths and mortality among drug users in Iran. 2020 (Unpublished data).

[15] Ma J, Bao YP, Wang RJ, Su MF, Liu MX, Li JQ (2019). Effects of medication-assisted treatment on mortality among opioids users: a systematic review and meta-analysis. Mol Psychiatry.

[16] Sordo L, Barrio G, Bravo MJ, Indave BI, Degenhardt L, Wiessing L (2017). Mortality risk during and after opioid substitution treatment: systematic review and meta-analysis of cohort studies. BMJ.

[17] Evans E, Li L, Min J, Huang D, Urada D, Liu L (2015). Mortality among individuals accessing pharmacological treatment for opioid dependence in California, 2006-10. Addiction.

[18] Hojjat S, Rezaei M, Mohamadipoor M, Norozi Km, Danesh M, Hatami S. The comparison of retention in three methods with methadone, opium and buprenorphine in patients admitted to addiction treatment centers. J North Khorasan Univ Med Sci 2016;8(2):245-56. [Persian].

[19] Hoseinie L, Gholami Z, Shadloo B, Mokri A, Amin-Esmaeili M, Rahimi-Movaghar A (2017). Drop-out from a drug treatment clinic and associated reasons. East Mediterr Health J.

[20] Sheikh Fathollahi M, Torkashvand F, Najmeddin H, Rezaeian M (2016). Predictors of one-year retention in methadone maintenance treatment (MMT) in Iran, Rafsanjan. Int J High Risk Behav Addict.

[21] Mohebi MD, Sargolzei N, Adibi A. Evaluation of retention in methadone treatment in patients attending Baharan hospital clinic in Zahedan city. Avicenna J Clin Med 2015;22(1):30-6. [Persian].

[22] Pashaei T, Moeeni M, Roshanaei Moghdam B, Heydari H, Turner NE, Razaghi EM (2014). Predictors of treatment retention in a major methadone maintenance treatment program in Iran: a survival analysis. J Res Health Sci.

[23] Kassani A, Niazi M, Hassanzadeh J, Menati R (2015). Survival analysis of drug abuse relapse in addiction treatment centers. Int J High Risk Behav Addict.

[24] Mattick RP, Breen C, Kimber J, Davoli M. Buprenorphine maintenance versus placebo or methadone maintenance for opioid dependence. Cochrane Database Syst Rev. 2014(2):CD002207. 10.1002/14651858.CD002207.pub4 PMC10617756 24500948

[25] Rahimi-Movaghar A, Amin-Esmaeili M, Hefazi M, Yousefi-Nooraie R. Pharmacological therapies for maintenance treatments of opium dependence. Cochrane Database Syst Rev. 2013(1):CD007775. 10.1002/14651858.CD007775.pub2 PMC11955260 23440817

[26] Wittchen HU, Apelt SM, Soyka M, Gastpar M, Backmund M, Gölz J (2008). Feasibility and outcome of substitution treatment of heroin-dependent patients in specialized substitution centers and primary care facilities in Germany: a naturalistic study in 2694 patients. Drug Alcohol Depend.

[27] Darke S, Ross J, Mills KL, Williamson A, Havard A, Teesson M (2007). Patterns of sustained heroin abstinence amongst long-term, dependent heroin users: 36 months findings from the Australian Treatment Outcome Study (ATOS). Addict Behav.

[28] Gossop M, Marsden J, Stewart D, Kidd T (2003). The National Treatment Outcome Research Study (NTORS): 4-5 year follow-up results. Addiction.

[29] Khodabandeh F, Kahani S, Shadnia S, Abdollahi M (2012). Comparison of the efficacy of methadone maintenance therapy vs. narcotics anonymous in the treatment of opioid addiction: a 2-year survey. Int J Pharmacol.

[30] Minozzi S, Amato L, Davoli M. Maintenance treatments for opiate dependent adolescent. Cochrane Database Syst Rev. 2009(2):CD007210. 10.1002/14651858.CD007210.pub2 19370679

[31] Johnson RE, Chutuape MA, Strain EC, Walsh SL, Stitzer ML, Bigelow GE (2000). A comparison of levomethadyl acetate, buprenorphine, and methadone for opioid dependence. N Engl J Med.

[32] Comiskey C, Kelly P, Leckey Y, McCullough L, O’duill B, Stapleton R, et al. The ROSIE Study: Drug Treatment Outcomes in Ireland. Stationery Office; 2009.

[33] Soyka M, Strehle J, Rehm J, Bühringer G, Wittchen HU (2017). Six-year outcome of opioid maintenance treatment in heroin-dependent patients: results from a naturalistic study in a nationally representative sample. Eur Addict Res.

[34] Mokri A, Chawarski MC, Taherinakhost H, Schottenfeld RS (2016). Medical treatments for opioid use disorder in Iran: a randomized, double-blind placebo-controlled comparison of buprenorphine/naloxone and naltrexone maintenance treatment. Addiction.

[35] Shadloo B, Baheshmat Sh, Rostam-Abadi Y, Shakeri A, Gholami J, Rahimi-Movaghar A. Comparison of Self-Reported Substance Use with Biological Testing among Treatment-seeking Patients with Opioid and Stimulant Use Disorders. 2020 (Unpublished data). 10.1016/j.jsat.2021.108555 34210569

[36] Farzam H, Farhadi KH, Rezaei M, Tolouei A. Comparing ultra rapid opiate detoxification with methadone in recurrent of self-introduced addicted subjects. J Kermanshah Univ Med Sci 2010;14(3):185-9. [Persian].

[37] Tatary F, Shakeri J, Nasiri A, Ghelichi L, Abdoli G. Naltrexone therapy and relapse rates of opioid dependent individuals. J Kermanshah Univ Med Sci 2007;10(3):332-41. [Persian].

[38] Ziyaodini H, Parvaresh N, Afshar N, Hoseiniyan SM, Sarhadi R, Hagdost AA. Comparison of the outcomes of three detoxification methods (clonidin, methadon, rapid) in opioid-dependents referred to Kerman Shaheed Beheshti hospital in a 6-month follow-up. J Kermanshah Univ Med Sci 2011;18(3):246-59. [Persian].

[39] Minozzi S, Amato L, Vecchi S, Davoli M, Kirchmayer U, Verster A (2011). Oral naltrexone maintenance treatment for opioid dependence. Cochrane Database Syst Rev.

[40] Rahimi-Movaghar A, Gholami J, Amato L, Hoseinie L, Yousefi-Nooraie R, Amin-Esmaeili M (2018). Pharmacological therapies for management of opium withdrawal. Cochrane Database Syst Rev.

[41] Jarvis BP, Holtyn AF, Subramaniam S, Tompkins DA, Oga EA, Bigelow GE (2018). Extended-release injectable naltrexone for opioid use disorder: a systematic review. Addiction.

[42] Zare H, Alipoor A, Aghamohammadhasani P, Nazer M, Mokhtaree M, Sayadi A (2012). Assessment role of participation in narcotic anonymous in opiate dependents during abstinence. Zahedan J Rese Med Sci.

[43] Aramideh Z, Sahbaeiroy F (2019). Sustained remission from drug addiction among the attendees of the meetings of anonymous addicts and rehabilitation centers in Mashhad, Iran, During 2017. Soc Behav Res Health.

[44] Akbari H, Roshanpajouh M, Nourijelyani K, Mansournia MA, Rahimi-Movaghar A, Yazdani K (2019). Profile of drug users in the residential treatment centers of Tehran, Iran. Health Promot Perspect.

[45] Morozova O, Crawford FW, Cohen T, Paltiel AD, Altice FL (2020). Cost-effectiveness of expanding the capacity of opioid agonist treatment in Ukraine: dynamic modeling analysis. Addiction.

[46] Degenhardt L, Grebely J, Stone J, Hickman M, Vickerman P, Marshall BDL (2019). Global patterns of opioid use and dependence: harms to populations, interventions, and future action. Lancet.

[47] Shield KD, Rehm J, Rehm MX, Gmel G, Drummond C (2014). The potential impact of increased treatment rates for alcohol dependence in the United Kingdom in 2004. BMC Health Serv Res.

[48] Mosadeghrad AM (2014). Factors affecting medical service quality. Iran J Public Health.

[49] Saloner B, Akosa Antwi Y, Maclean JC, Cook B (2018). Access to health insurance and utilization of substance use disorder treatment: evidence from the Affordable Care Act dependent coverage provision. Health Econ.

[50] Rahimi-Movaghar A, Amin-Esmaeili M, Shadloo B, Noroozi A, Malekinejad M (2015). Transition to injecting drug use in Iran: a systematic review of qualitative and quantitative evidence. Int J Drug Policy.

[51] Malekinejad M, Vazirian M (2012). Transition to injection amongst opioid users in Iran: implications for harm reduction. Int J Drug Policy.

