# Supplementary material for: Opium dependence and the potential impact of changes in treatment coverage level: A dynamic modeling study
Source: Health Promot Perspect. 2021 May 19;11(2):240–9. doi: 10.34172/hpp.2021.29 (PMC8233677; doi:10.34172/hpp.2021.29)
Supplement: Supplementary file 1 — Estimation of model parameter’s values. [file hpp-11-240-s001.pdf]

Opium Dependence, the Potential Impact of Changes in Treatment Coverage Level: A Dynamic Modeling Study

Supplementary file 1. Estimation of model parameter's values

In this section, the calculations and how to estimate each of the parameters are explained in detail.

- **Opium dependent population in Iran and the of each treatment group's entry to percentage**

The annual entry to detoxification treatment based on secondary analysis of TEDS[1] data and RSA report[2] was 11.1% and 8.9%, respectively. So, the share of detoxification and relapse prevention treatment (except NA treatment) was considered 5% of the total treatment, based on the experts' consensus about the population of opium dependence throughout the country. We also considered NA group within this group and according to RSA, 37.1 percent of opioid substance dependence had experienced some types of treatment and 12 steps (NA) groups accounted for 14% of treatment experienced people. In other words, 5 percent of all opium substance dependence. According to a study conducted in Mashhad, Iran, about 50% of patients in the NA group were opium dependence,[3] while this amount for whole country is 70% of all addictions (RSA study), so with regard to this ratio and consensus of experts, NA entry percentage after detoxification was considered equal to 3.5% of all opium dependence in Iran. So, coverage of detoxification and relapse prevention treatment was considered 8.5% of the total treatment and this amount will be 2.42% for the whole population.

The ratio of MMT to BMT maintenance treatment entrance for opium dependence was 79.42 vs. 20.58 based on new cases data of IDATIS for 2019,[4] and this amount was also agreed upon by experts and was considered in the model.

The evidence-based medicine treatment coverage was 27.5% based on the results of TEDS. Also, in the National Mental Health Survey,[5] 25% of opioid dependence had the experience of referring to outpatient treatment centers. In the RSA study, about 55% of all opioid dependence experienced treatment in one of the maintenance or drug detoxification groups. Which according to expert's opinion, this percentage for those "opioid dependents that were not recruited from any service settings" is at least half of all addicts, so in this case we will reach 27.5% of treatment coverage.

Based-on RSA study, the experience all types of treatment (evidence-based and non-evidence-based medicine) was 37% for the participants, while for outpatients and medium-term residential drug treatment centers it was 100% and for those not recruited from any service settings, it was 15.8%.

- **Prevalence of opium dependence in Iran**

In the IranMHS study[5] (which was a household survey), the prevalence of 12-month opioid substances use disorder was 2.23% based-on DSM-5. Considering that 84% of these cases were related to opium dependence and its derivatives, the prevalence of opium dependence for the population aged 15-64 years was 1.87%. On the other hand, according to experts and considering 30% underestimation which arises due to the social stigma of addiction and the nature of household survey studies, and 20% overestimation due to considering the mild cases in DSM-5, the final prevalence of Opium dependence was estimated 2.06% for the population of 15 -64 years old in Iran.

- **Heroin dependence following Opium dependence**

The results of a longitudinal study,[6] on changes in the consumption pattern of 70 patients with soft opioid substances dependence (including opium, SHIREH and prescription opioids) showed that 5.7% of patients (95% CI: 2.1 to 14.6) were changed to hard opioid users (including heroin or crack heroin) after 6 years. On the other hand, in the secondary analysis of TEDS data, heroin use prevalence in opioid dependence who had referred to treatments was 2.68% per year. Based on these results and the consensus of experts, the annual percentage of heroin dependence following opium dependence was considered equal to 1.5%.

- **Annual mortality from opium dependence**

According to the results of INCAS systematic review and meta-analysis study of mortality of Iranian drug dependence, the crude death rate in opioid dependence is reported to be 1.52% per year.[7] (Table S1 and Figure S1). Excluding the study of Nazer, 2008[8] and Rahimi-Movaghar A., 2011[9] (due to the study of more high-risk addicts) the crude death rate was 1.15% per year (95% CI: 0.27 to 2.03) based on the meta-analysis of the remaining three studies[10-12] (Figure S1). Based on the consensus of experts, this death rate was considered as the total death in the opium dependence in Iran.

In the meta-analysis of international studies, the all causes mortality rate for the non-heroin non-injection opioid dependence group was 1.19% per year. Based on meta-analysis, treatment group's annual mortality rate for the methadone and buprenorphine maintenance treatment was 0.80 and 0.40, respectively (Figure S2). In this group of addicts (non-heroin, non-injection group), the mortality ratio of MMT and BMT groups to total mortality rate was 0.67 and 0.34, respectively. Considering this ratio for the total annual mortality rate of Iranian opium dependence (1.15% per year), the death rates for MMT and BMT groups of Iran were 0.77 and 0.39, respectively.

The mortality ratio of the detoxification and relapse prevention treatment group to total was estimated to be 0.46 based on the Evans, E. study.[13] The mortality rate in this treatment group for the Iranian opium dependence was 0.53% per year, based on ratio and total opium dependence mortality rate. The total mortality rate equation was consisted of four parts (3 treatment groups and one untreated or non-evidence-based medicine treatment group) and the only unknown mortality rate was untreated groups, which was calculated using available information and obtained 1.34% (Table S2).

- **Percentage of annual retention in maintenance treatments**

Annual retention in MMT for opioid dependence has been reported to be between 34% and 44% in the majority of reviewed Iranian studies.[14-20] According to the literature review and discussion in the panel of experts, this amount was estimated 33.3% per year for Iranian opium dependence. Also, according to Mattick (2014) meta-analysis of 11 studies, the ratio of BMT to MMT retention was 0.83,[21] which was agreed to be 0.9 for the Iranian opium dependence treatment. Therefore, the annual retention rate in BMT treatment was considered to be 0.30%.

- **Treatment success in each treatment group**

12-month abstinence rate in maintenance treatment is reported from 4 to 47% in various studies. However, in most of the studies, this rate was reported to be about 15%.[22-30] Since these studies have been conducted on both opioid and heroin dependence, it can be expected that this amount to be higher when considering opium dependence separately. According to another INCAS study, of the 159 opioid dependence who had been on maintenance treatment for more than a year, three had a

positive urine test, and five reported self-reported recent drug use. In other words, a maximum of 3% of people remain in treatment (failed to abstinence).[31] Since in this study, urine test was performed only once and also a considerable percentage of people were in treatment for more than a year, we expect the failure rate to be higher at the end of first year. Finally, after expert discussion, the percentage of abstinence at the end of one year was estimated at 27% and 26% for MMT and BMT, respectively.

The success of treatment (abstinence) in the detoxification group plus oral naltrexone in three Iranian studies was 32.8%[32], and 43.6%[33] at one year follow-up, and 83.3%[34] for six-month follow-up. In two Iranian 12-month follow-up studies about the success of treatment of NA group, the success rate was reported 28%[25] and 33%[35], and in another study, this value was equal to 34% for 6 months.[3] Due to the fact the fact that all above mentioned studies were conducted in a controlled condition and limited special medical clinics in Iran, and with regard to the available documents and the result of the FGD, the abstinence rate of detoxification and relapse prevention treatment group (psychological, NA group, oral and extended-release (XR) injection naltrexone) for opium dependence in Iran was estimated at 10%.

Table S1. Characteristics of the included studies on mortality among drug users in Iran

Study ID (First author, year of publication, and language); Reference	Study design	Province	Target Population	Opioid use	Sampling sites	Sampling method	Sample size (Male, Female)
Rahimi-Movaghar, unpublished data of youth cohort, 2019 (personal contact)	Cohort study	Fars	15-34 years old residents who has positive history of any opioid use in last 12 months at baseline	Opium99.2%; Heroin 4%; Tramadol 14.8%; Illicit Buprenorphine or methadone 31.18%	General population of Fasa and Ravansar	Cluster random sampling of households	263 (235,28)
Khademi 2012, English;	Prospective cohort	Golestan	Opium users aged 40-75	100% Opium	Household	Urban: random systematic clustering Rural: census	8487 (6132, 2355)
Rahimi-Movaghar, 2011, Persian;	Follow-up study	Tehran	Street addicts who were arrested for mandatory treatment of addiction (Nejat program)	Opium 86.3%; Heroin 54.1%; Tramadol 20.5%; Buprenorphine 5.6%	Valiasr garrison	Census	497 (495,2)
Nazer, 2008, English (Congress Abstract)	Retrospective cohort	Lorestan	Subjects enrolled in the drug treatment program (HIV positive, negative or untested)	UK	Treatment facilities	Census	2996 (UK) (814 HIV positive, 10635HIV negative, 1119 untested)
Jafari, 2010, English	Follow-up study	Fars	Drug users referred for treatment of opium, heroin or buprenorphine dependence	Opium 99%; Heroin 9%; buprenorphine 24%	Treatment facility	UK	273 (UK)

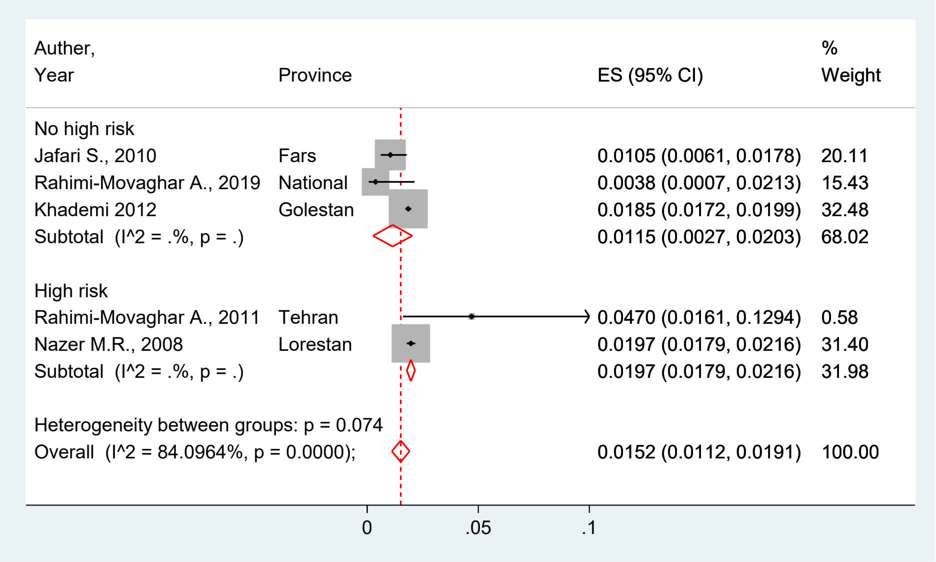

Figure S1. Crude death rate (per 100-person year) for opioid dependence in Iran, based on the INCAS systematic review by high-risk and non-high-risk (representative of opium dependence)

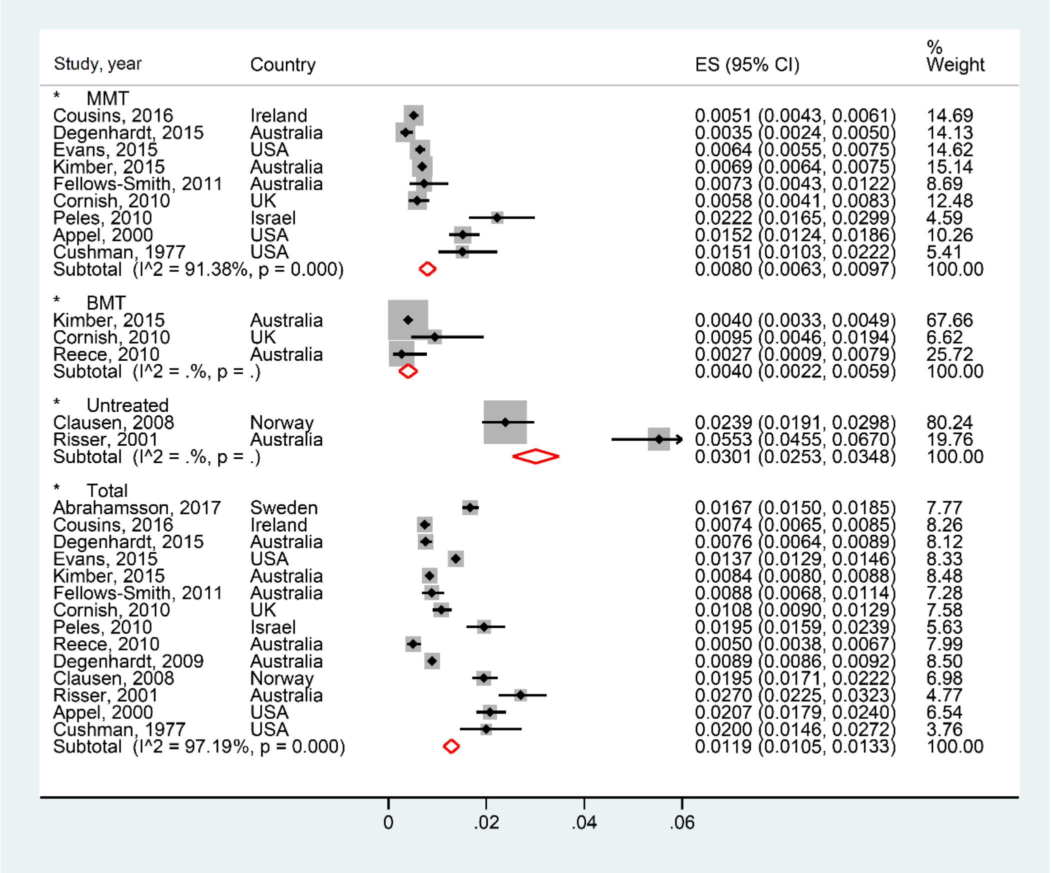

Figure S2. Crude death rate (per 100-person year) for the non-heroin non-injection opioid dependence by treatment group

Table S2. Estimation of mortality rate of different groups of opium dependence

In treatment group of non-heroin, non-injecting opioid dependent	Meta-analysis result			Death rate ratio of treatment group to total dependence	Estimation of annual mortality rate for subgroups of opium dependence in Iran (%)
	Number of articles	Crude death rate	References		
**In MMT**	9	0.80 (0.63 to 0.97)	[13, 36-43]	0.67	0.77 [a]
**In BMT**	3	0.40 (0.22 to 0.59)	[37, 42, 44]	0.34	0.39 [a]
**In untreated**	2	3.01 (2.53 to 3.48)	[45, 46]	2.53	1.34 [b]
**Total dependence**	14	1.19 (1.05 to 1.33)	[13, 36-48]	1.0	1.15

a. Based-on the ratio of death rate in subgroups to death rate in total addicts obtained from meta-analysis of international studies
b. Calculation based-on the total death rate in Iran and taking into account the death rate for other treatment groups

References

1. INCAS-TEDS. Iranian National Center for Addiction Studies (INCAS) Treatment Episode Dataset: TEDS [Unpublished data]. 2017-2019.
2. Rafiey H. MS, Narenjiha H., Alipour F., . Rapid Situation Assessment of Drug Abuse in Iran, 2018 [Unpublished data]. Department of Research and Education, Drug Control Headquarters, Presidency of the IR of Iran. 2020.
3. Aramideh Z, Sahbaeiroy F. Sustained Remission from Drug Addiction among the Attendees of the Meetings of Anonymous Addicts and Rehabilitation Centers in Mashhad, Iran, During 2017. Social Behavior Research Health. 2019; 3(2): 378-84.
4. IDATIS. Iran Substance Abuse Treatment Information System.
5. Amin-Esmaeili M, Rahimi-Movaghar A, Sharifi V, Hajebi A, Radgoodarzi R, Mojtabai R, et al. Epidemiology of illicit drug use disorders in Iran: prevalence, correlates, comorbidity and service utilization results from the Iranian Mental Health Survey. Addiction. 2016; May 14. doi: 10.1111/add.13453.
6. INCAS. Outcome of opioid use and opioid use disorder; a six-year follow-up [Unpublished data]. 2019.
7. Gholami J. et al. Drug-related deaths and mortality among drug users in Iran [Unpublished data]. 2020.
8. Nazer MR. Mortality and Death among HIV Positive Injection Drug Users [Congress Abstract]. The Second Annual Conference on AIDS Research in Iran; Tehran2008.
9. Rahimi-Movaghar A, Khsto GA, Razagi E, Saberi-Zafargandi MB, Noroozi A, Jarsiyah R. Compulsory treatment of methadone addicts in a residential center (2): evaluation of results in two- and six-month follow-up [In persian]. Payesh. 2011; 10(4): -.
10. Khademi H, Malekzadeh R, Pourshams A, Jafari E, Salahi R, Semnani S, et al. Opium use and mortality in Golestan Cohort Study: prospective cohort study of 50,000 adults in Iran. Bmj. 2012; 344: e2502. doi:10.1136/bmj.e2502
11. Jafari S, Rahimi-Movaghar A, Craib KJ, Baharlou S, Mathias R. A follow-up study of drug users in Southern Iran. Addiction Research & Theory. 2010; 18(1): 59-70.
12. Rahimi-Movaghar A, Amin-Esmaeili M, Sharifi V, Hajebi A, Motevalian S. Data from Persian Youth Cohort. [Unpublished].

13. Evans E, Li L, Min J, Huang D, Urada D, Liu L, et al. Mortality among individuals accessing pharmacological treatment for opioid dependence in California, 2006-10. Addiction. 2015; 110(6): 996-1005. doi:10.1111/add.12863
14. Hoseinie L, Gholami Z, Shadloo B, Mokri A, Amin-Esmaeili M, Rahimi-Movaghar A. Drop-out from a drug treatment clinic and associated reasons. East Mediterr Health J. 2017; 23(3): 173-81. doi:10.26719/2017.23.3.173
15. Sheikh Fathollahi M, Torkashvand F, Najmeddin H, Rezaeian M. Predictors of One-Year Retention in Methadone Maintenance Treatment (MMT) in Iran, Rafsanjan. Int J High Risk Behav Addict. 2016; 5(3): e29121. doi:10.5812/ijhrba.29121
16. Mohebi MD, Sargolzei N, Adibi A. Evaluation of Retention in Methadone Treatment in Patients Attending Baharan Hospital Clinic in Zahedan City. Avicenna Journal of Clinical Medicine. 2015; 22(1): 30-6.
17. Pashaei T, Moeeni M, Roshanaei Moghdam B, Heydari H, Turner NE, Razaghi EM. Predictors of treatment retention in a major methadone maintenance treatment program in iran: a survival analysis. J Res Health Sci. 2014; 14(4): 291-5.
18. Hojjat S, Rezaei M, mohamadipoor M, NOROZI KM, Danesh M, Hatami S. The comparison of Retention in three methods with Methadone, opium and Buprenorphine in patients admitted to addiction treatment centers [In persian] Journal of North Khorasan University of Medical Sciences. 2016; 8(2).
19. Kassani A, Niazi M, Hassanzadeh J, Menati R. Survival Analysis of Drug Abuse Relapse in Addiction Treatment Centers. Int J High Risk Behav Addict. 2015; 4(3): e23402. doi:10.5812/ijhrba.23402
20. Rahimi-Movaghar A, Amin-Esmaeili M, Hefazi M, Yousefi-Nooraie R. Pharmacological therapies for maintenance treatments of opium dependence. Cochrane Database Syst Rev. 2013; (1): Cd007775. doi:10.1002/14651858.CD007775.pub2
21. Mattick RP, Breen C, Kimber J, Davoli M. Buprenorphine maintenance versus placebo or methadone maintenance for opioid dependence. Cochrane Database Syst Rev. 2014; (2): Cd002207. doi:10.1002/14651858.CD002207.pub4
22. Wittchen HU, Apelt SM, Soyka M, Gastpar M, Backmund M, Gölz J, et al. Feasibility and outcome of substitution treatment of heroin-dependent patients in specialized substitution centers and primary care facilities in Germany: a naturalistic study in 2694 patients. Drug Alcohol Depend. 2008; 95(3): 245-57. doi:10.1016/j.drugalcdep.2008.01.015
23. Darke S, Ross J, Mills KL, Williamson A, Havard A, Teesson M. Patterns of sustained heroin abstinence amongst long-term, dependent heroin users: 36 months findings from the Australian Treatment Outcome Study (ATOS). Addict Behav. 2007; 32(9): 1897-906. doi:10.1016/j.addbeh.2007.01.014
24. Gossop M, Marsden J, Stewart D, Kidd T. The National Treatment Outcome Research Study (NTORS): 4-5 year follow-up results. Addiction. 2003; 98(3): 291-303. doi:10.1046/j.1360-0443.2003.00296.x
25. Khodabandeh F, Kahani S, Shadnia S, Abdollahi M. Comparison of the efficacy of methadone maintenance therapy vs. narcotics anonymous in the treatment of opioid addiction: A 2-year survey. International Journal of Pharmacology. 2012; 8(5): 445-9.
26. Minozzi S, Amato L, Davoli M. Maintenance treatments for opiate dependent adolescent. Cochrane Database Syst Rev. 2009; (2): Cd007210. doi:10.1002/14651858.CD007210.pub2
27. Johnson RE, Chutuape MA, Strain EC, Walsh SL, Stitzer ML, Bigelow GE. A comparison of levomethadyl acetate, buprenorphine, and methadone for opioid dependence. N Engl J Med. 2000; 343(18): 1290-7. doi:10.1056/nejm200011023431802
28. Comiskey C, Kelly P, Leckey Y, McCullough L, O'duill B, Stapleton R, et al. The ROSIE study: Drug treatment outcomes in Ireland: Stationery Office; 2009. p.
29. Soyka M, Strehle J, Rehm J, Bühringer G, Wittchen HU. Six-Year Outcome of Opioid Maintenance Treatment in Heroin-Dependent Patients: Results from a Naturalistic Study in a Nationally Representative Sample. Eur Addict Res. 2017; 23(2): 97-105. doi:10.1159/000468518
30. Mokri A, Chawarski MC, Taherinakhost H, Schottenfeld RS. Medical treatments for opioid use disorder in Iran: a randomized, double-blind placebo-controlled comparison of buprenorphine/naloxone and naltrexone maintenance treatment. Addiction. 2016; 111(5): 874-82. doi:10.1111/add.13259
31. Shadloo B. et al. Comparison of Self-Reported Substance Use with Biological Testing among Treatment-seeking Patients with Opioid and Stimulant Use Disorders [Unpublished data]. 2020.
32. Tatary F, Shakeri J, Nasiri A, Ghelichi L, Abdoli G. Naltrexone therapy and relapse rates of opioid dependent individuals [In persian]. J Kermanshah Univ Med Sci. 2007; 10(3): 332-40.
33. Farzam H, Farhadi K, Tolouei AR, Rezaei M. Comparing Ultra Rapid Opiate Detoxification with Methadone in Recurrent of Self-Introduced Addicted Subjects [In persian]. Journal of Kermanshah University of Medical Sciences. 2010; 14(3): 185-9.
34. Ziyaodini H, Parvaresh N, Afshar N, Hoseiniyan S, Sarhadi R, Hagdost A. Comparison of the outcomes of three detoxification methods (Clonidin, methadon, rapid) in opioid-dependents referred to kerman

shaheed beheshti hospital in a 6-month follow-up [In persian]. Journal of Kerman University of Medical Sciences. 2011; 18(3): 246-59.
35. Zare H, Alipoor A, Aghamohammadhasani P, Nazer M, Mokhtaree M, Sayadi A. Assessment role of participation in narcotic anonymous in opiate dependents during abstinence. Zahedan J Rese Med Sci. 2012; 14(9): 42-6.
36. Appel PW, Joseph H, Richman BL. Causes and rates of death among methadone maintenance patients before and after the onset of the HIV/AIDS epidemic. Mt Sinai J Med. 2000; 67(5-6): 444-51.
37. Cornish R, Macleod J, Strang J, Vickerman P, Hickman M. Risk of death during and after opiate substitution treatment in primary care: prospective observational study in UK General Practice Research Database. Bmj. 2010; 341: c5475. doi:10.1136/bmj.c5475
38. Cousins G, Boland F, Courtney B, Barry J, Lyons S, Fahey T. Risk of mortality on and off methadone substitution treatment in primary care: a national cohort study. Addiction. 2016; 111(1): 73-82. doi:10.1111/add.13087
39. Cushman P, Jr. Ten years of methadone maintenance treatment: some clinical observations. Am J Drug Alcohol Abuse. 1977; 4(4): 543-53. doi:10.3109/00952997709007010
40. Degenhardt L, Randall D, Hall W, Law M, Butler T, Burns L. Mortality among clients of a state-wide opioid pharmacotherapy program over 20 years: risk factors and lives saved. Drug Alcohol Depend. 2009; 105(1-2): 9-15. doi:10.1016/j.drugalcdep.2009.05.021
41. Fellows-Smith J. Opioid-dependent error processing. J Opioid Manag. 2011; 7(6): 443-9.
42. Kimber J, Larney S, Hickman M, Randall D, Degenhardt L. Mortality risk of opioid substitution therapy with methadone versus buprenorphine: a retrospective cohort study. Lancet Psychiatry. 2015; 2(10): 901-8. doi:10.1016/s2215-0366(15)00366-1
43. Peles E, Schreiber S, Adelson M. 15-Year survival and retention of patients in a general hospital-affiliated methadone maintenance treatment (MMT) center in Israel. Drug Alcohol Depend. 2010; 107(2-3): 141-8. doi:10.1016/j.drugalcdep.2009.09.013
44. Reece AS. Favorable mortality profile of naltrexone implants for opiate addiction. J Addict Dis. 2010; 29(1): 30-50. doi:10.1080/10550880903435988
45. Clausen T, Anchersen K, Waal H. Mortality prior to, during and after opioid maintenance treatment (OMT): a national prospective cross-registry study. Drug Alcohol Depend. 2008; 94(1-3): 151-7. doi:10.1016/j.drugalcdep.2007.11.003
46. Risser D, Hönigschnabl S, Stichenwirth M, Pfudl S, Sebald D, Kaff A, et al. Mortality of opiate users in Vienna, Austria. Drug Alcohol Depend. 2001; 64(3): 251-6. doi:10.1016/s0376-8716(01)00131-4
47. Abrahamsson T, Berge J, Öjehagen A, Håkansson A. Benzodiazepine, z-drug and pregabalin prescriptions and mortality among patients in opioid maintenance treatment-A nation-wide register-based open cohort study. Drug Alcohol Depend. 2017; 174: 58-64. doi:10.1016/j.drugalcdep.2017.01.013
48. Degenhardt L, Larney S, Kimber J, Farrell M, Hall W. Excess mortality among opioid-using patients treated with oral naltrexone in Australia. Drug Alcohol Rev. 2015; 34(1): 90-6. doi:10.1111/dar.12205
